# Web-Based Activity Within a Sexual Health Economy: Observational Study

**DOI:** 10.2196/jmir.8101

**Published:** 2018-03-07

**Authors:** Katy ME Turner, Adam K Zienkiewicz, Jonathan Syred, Katharine J Looker, Joia de Sa, Michael Brady, Caroline Free, Gillian Holdsworth, Paula Baraitser

**Affiliations:** ^1^ Bristol Veterinary School University of Bristol Bristol United Kingdom; ^2^ Population Health Sciences Bristol Medical School University of Bristol Bristol United Kingdom; ^3^ King's Center for Global Health and Health Partnerships King's College London London United Kingdom; ^4^ School of Population Health & Environmental Sciences King's College London London United Kingdom; ^5^ School of Population Health Sciences Bristol Medical School University of Bristol Bristol United Kingdom; ^6^ SH:24 London United Kingdom; ^7^ Department of Sexual Health and HIV Kings College Hospital NHS Foundation Trust London United Kingdom; ^8^ Department of Population Health London School of Hygiene and Tropical Medicine London United Kingdom; ^9^ King's Center for Global Health and Health Partnerships School of Population Health & Environmental Sciences King's College London London United Kingdom

**Keywords:** sexually transmitted diseases, testing, internet, self-sampling

## Abstract

**Background:**

Regular testing for sexually transmitted infections (STIs) is important to maintain sexual health. Self-sampling kits ordered online and delivered in the post may increase access, convenience, and cost-effectiveness. Sexual health economies may target limited resources more effectively by signposting users toward Web-based or face-to-face services according to clinical need.

**Objective:**

The aim of this paper was to investigate the impact of two interventions on testing activity across a whole sexual health economy: (1) the introduction of open access Web-based STI testing services and (2) a clinic policy of *triage and signpost* online where users without symptoms who attended clinics for STI testing were supported to access the Web-based service instead.

**Methods:**

Data on attendances at all specialist public sexual health providers in an inner-London area were collated into a single database. Each record included information on user demographics, service type accessed, and clinical activity provided, including test results. Clinical activity was categorized as a simple STI test (could be done in a clinic or online), a complex visit (requiring face-to-face consultation), or other.

**Results:**

Introduction of Web-based services increased total testing activity across the whole sexual health economy by 18.47% (from 36,373 to 43,091 in the same 6-month period—2014-2015 and 2015-2016), suggesting unmet need for testing in the area. Triage and signposting shifted activity out of the clinic onto the Web-based service, with simple STI testing in the clinic decreasing from 16.90% (920/5443) to 12.25% (511/4172) of total activity, *P*<.001, and complex activity in the clinic increasing from 69.15% (3764/5443) to 74.86% (3123/4172) of total activity, *P*<.001. This intervention created a new population of online users with different demographic and clinical profiles from those who use Web-based services spontaneously. Some triage and signposted users (29.62%, 375/1266) did not complete the Web-based testing process, suggesting the potential for missed diagnoses.

**Conclusions:**

This evaluation shows that users can effectively be transitioned from face-to-face to Web-based services and that this introduces a new population to Web-based service use and changes the focus of clinic-based activity. Further development is underway to optimize the triage and signposting process to support test completion.

## Introduction

Regular testing for sexually transmitted infections (STIs) with rapid treatment and partner notification are important strategies to improve and maintain sexual health [1]. Testing for chlamydia, gonorrhea, HIV, and syphilis is traditionally delivered within sexual health clinics, but Web-based testing is increasingly part of the sexual health economy [2-5]. This reflects the English National Health Service (NHS) strategy on digital care to meet expanding health care demand within limited resources [6,7].

Web-based STI testing services may provide self-tests where users both collect samples and read the results, or self-sampling where users collect samples that are sent to the laboratory for processing [8]. They offer 24-hour access to testing without the need to visit a clinic and may facilitate effective use of clinic services by shifting simple testing (testing with no other clinical activity required) online and freeing clinic capacity for complex care [9]. Although there is increasing availability of internet-based ordering of STI tests in developed countries, such services are highly heterogeneous: some are targeted to specific risk groups (based on age, ethnicity, or sexual orientation), test for a single infection [10], may be fully integrated with existing health services [11] or completely independent. A randomized controlled trial in France reported an increase in testing uptake (29.2% in the intervention group vs 8.7% in the control group, risk ratio: 3.37, 95% CI 3.05-3.74) [12]. However, outcomes were assessed using different measures in the intervention and control group, and there was low follow-up [12]. One US study found that internet-based testing could facilitate testing of high-risk individuals who were not accessing clinic-based services [13].

This paper evaluates the impact of Web-based self-sampling services within a sexual health economy within the London Boroughs of Lambeth and Southwark, an inner London area, with high rates of sexual ill health [14]. Unmet need was present before the introduction of an Web-based service, with 17,000 people having turned away from all sexual health services in the area annually (local clinic data) because of insufficient clinical capacity to meet demand. The Web-based service (SH:24 [15]) in this area provides free access to testing for chlamydia, gonorrhea, HIV, and syphilis for users older than 16 years with no restrictions based on gender or sexual orientation. Users complete an order form with self-sampling kits delivered home. Test kits are tailored to gender and sexuality. They include written information and link to a video that explains the self-sampling process. Participants can text or request a call-back for questions or concerns. Nonreturners are sent reminders via an SMS text message (short message service, SMS) and additional test kits if required. Results are sent by SMS text message except HIV reactive results that are delivered by telephone. At the time of this study, all those with positive results are referred into clinics for treatment and partner notification and managed according to national guidelines. Notification (by SMS text message) and management (treatment and partner notification) of patients identified through online self-sampling is the same as for asymptomatic patients tested in the clinic.

This paper documents STI testing before and after the introduction of online self-sampling services available to those aged 16 years or above and resident in the area. It documents the impact of a subsequent change in policy at one clinic—Camberwell Sexual Health Service, a large service providing both contraception and diagnosis and management of STIs. The change in policy was designed to facilitate the transition of simple STI testing from the clinic to the online service. This is an emerging strategy, developed in response to the online service. The new clinic policy introduced a triage process managed by clinical support workers who assess clinical need and redirect asymptomatic users requesting STI testing alone to order their tests via the online service using tablets available in the clinic. After online ordering, clinic staff prepare the self-sampling packs immediately for users to take away. Users then follow the online self-sampling process described above. The policy is designed to release capacity within the clinical service and use this for the management of complex clinical need. It is also intended to change future user behavior so that the online service becomes a first choice for future testing. The evaluation presented here analyses the impact of these service developments on sexual health activity across the whole sexual health system.

The aim of this observational study was to investigate the effect of the real life resource allocation decisions made within clinics following the establishment of an effective online service. This will inform policy makers and commissioners about the potential impact of changes to service capacity and delivery.

## Methods

### Data Sources and Preparation

**Figure 1:**
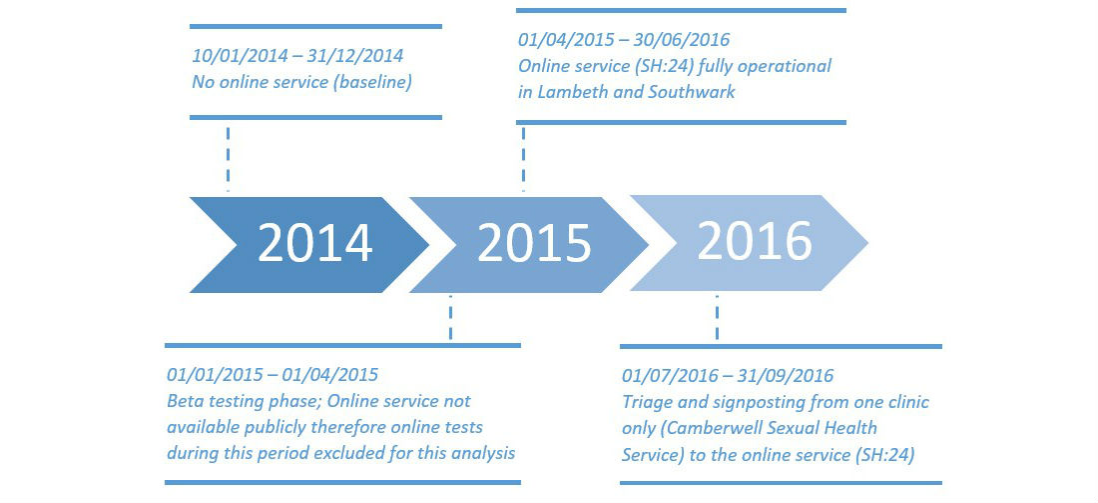
Timeline of sexual health service changes in Southwark and Lambeth from 2014 to 2016.

Records of clinic visits for all sexual health attendances in the London Boroughs of Lambeth and Southwark were collated from January 1, 2014 to September 31, 2016 from all sexual health service providers: genitourinary medicine (GUM) clinics, integrated clinics, community sexual and reproductive health clinics, and the online service. This covered a baseline period where there were no major changes to sexual health service provision, followed by implementation of online STI testing, and then changes in clinic practice resulting from availability of the new service (Figure 1).

All records were anonymized to remove identifiable information and harmonized to generate a complete dataset of individual level clinic attendances (one record, per person, per day). Each record includes demographic information: unique user identification number, gender, age at visit, site of visit, ethnicity, area of residence (lower super output area code), sexual orientation, and clinical information (first or follow-up visits and up to 12 sexual health, six reproductive health, and five contraception method *codes* for clinic activity). Individual level clinic attendance data were collated and summarized as simple STI test performed (chlamydia, gonorrhea, HIV, and syphilis) or complex service required. Area of residence was summarized as Lambeth, Southwark, adjacent boroughs, other London, or out of London. We considered the impact of the changes on Lambeth and Southwark residents only as access to the online service was restricted to this group. Clinic activity was coded using the GUM clinic activity dataset version 2 codes (Multimedia Appendix 1), assigned by clinicians during or after the consultation. For clinic users, we assumed that symptoms were present if microscopy was recorded as an activity. Online users were directly asked a question about presence of symptoms by the online registration system, with advice to go to a clinic if symptoms were present. This question was worded as follows:

Are you showing any symptoms of a sexually transmitted infections? If you have symptoms, we advise that you go to a clinic. You can find more information about your local clinic here. You can find out more information on symptoms of sexually transmitted infections here. (additional information screen appears on click).

### Exclusion Criteria

Records were excluded from analysis if there were no codes associated with the clinic visit, or if individuals were prisoners, or younger than 16 years, or 100 years and older.

### Definition of Attendance Types and Positivity

Individual level clinic activity data were collated and summarized as “simple STI test” (chlamydia, gonorrhea, HIV, and syphilis) or “complex visit” (Multimedia Appendix 1). By definition, all Web activity was a simple STI test. We identified both attendances where a simple STI test was provided, as well as the subset of attendances where *only* a simple STI test was provided. A complex visit could be defined as an examination or physical intervention being recorded (eg, surgical, vaccination, or gynecological) or the patient was symptomatic.

The positivity was calculated as the number of positive diagnoses or total test records, both for all simple STI tests and for each of the four infections (gonorrhea, chlamydia, HIV, and syphilis) separately; further details of the calculation of positivity are given in Multimedia Appendix 1, equation 1.

### Ethics

Ethical approval was obtained from the NRES Committee North of Scotland—Grampian (Ref 15/NS/0031).

### Data Analysis

The overall pattern of STI testing across Lambeth and Southwark across all sexual health providers from January 1, 2014 to September 30, 2016 was analyzed. Linear regression was used to test for trend in testing volume at Camberwell Sexual Health Centre up to June 30, 2016. The complexity of clinic activity provided to Lambeth and Southwark residents at Camberwell Sexual Health Centre in 2016 during quarter 2 (Q2) was compared with that during quarter 3 (Q3). The triage and signposting service was introduced at the start of Q3. Changes in pattern of clinic attendance between the quarters were analyzed using a chi-square test. Test completion and positivity were evaluated for those who used the triage and signposting pathway. Populations who used the triage and signposting pathway in Q3 were compared with those residents in the same area accessing the Web-based service without signposting or triage (spontaneous users).

## Results

### Patterns of Sexually Transmitted Infection Testing in Southwark and Lambeth Over Time

**Figure 2:**
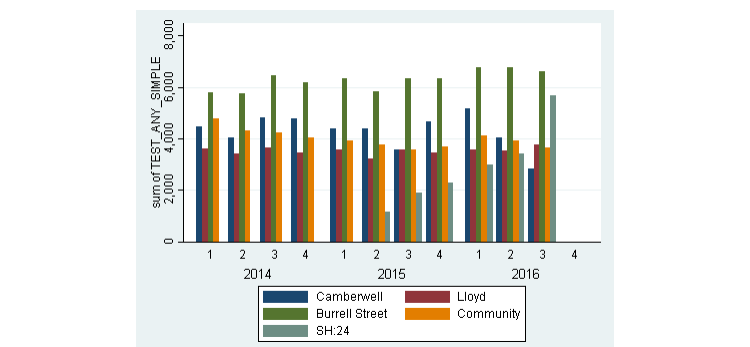
Number of simple sexually transmitted infections (STI) tests delivered across the whole sexual health economy, by service provider, by quarter, from quarter 1 (Q1) 2014 to quarter 3 (Q3) 2016 in Lambeth and Southwark, London.

**Figure 3:**
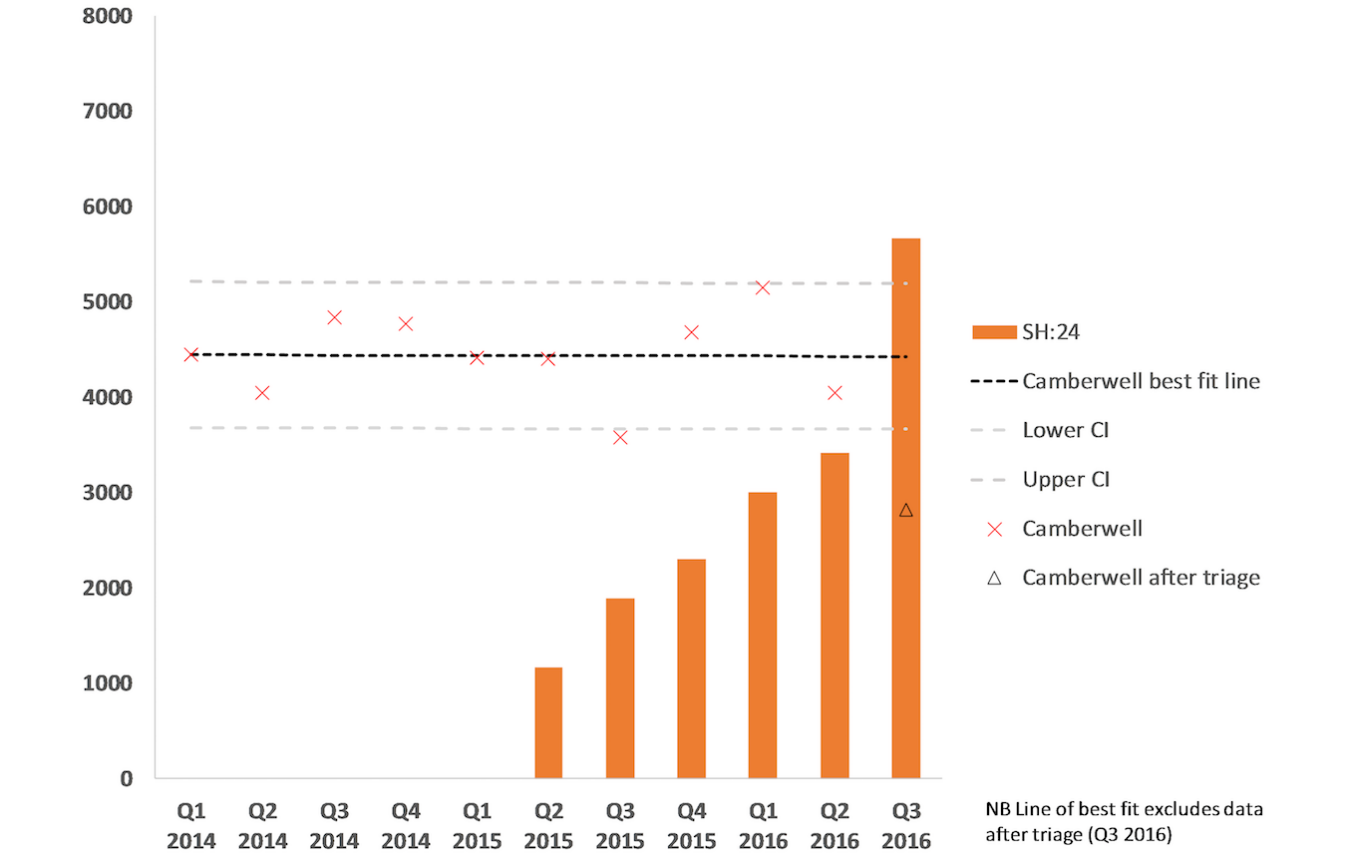
Number of simple sexually transmitted infections (STI) tests performed at Camberwell Sexual Health Centre and SH:24 by calendar year and quarter, quarter 1 (Q1) 2014 to quarter 3 (Q3) 2016.

The overall testing capacity across the whole sexual health economy remained stable during the 2 years before the introduction of Web-based testing and is shown in Figure 2. The addition of online services was associated with an increase in total testing across the whole sexual health economy gradually from its introduction in Q2, 2015 to peak activity in Q3, 2016 (Figure 3). In a 6-month period before online testing (October 1, 2014 to March 31, 2015), there were a total of 36,673 STI tests performed within Lambeth and Southwark. In the same period in 2015-2016, this increased by 18.47% to 43,091 tests. Before the introduction of triage and signposting from January 1, 2014 to June 30, 2016, there was no significant change in the number of STIs tests each quarter over time in Camberwell Sexual Health clinic, test for trend *P*=.97 (Figure 3) *.*

We compared clinic activity at Camberwell Sexual Health Service, following the introduction of triage and signposting to direct asymptomatic users in the clinic to the Web-based service, implemented from July 1, 2016 (Table 1). The introduction of triage and signposting was associated with a decrease in total activity in the clinic of 23% between Q2 (6946) and Q3 (5362). The total number of visits by Lambeth and Southwark resident that included an STI test decreased from 3156/5443 (57.98% of visits) in Q2 to 2202/4172 (52.78% of visits) in Q3. During the same period, the proportion of simple STI tests without additional complex activity decreased from 16.90% (920/5443) to 12.25% (511/4172; chi-square, *P*<.001), and the proportion of complex service activity increased from 69.15% (3764/5443) to 74.86% (3123/4172; chi-square, *P*<.001; Figure 3).

### Analysis of Sexually Transmitted Infection Testing Patterns in Quarter 3, 2016 Online and in Clinic

In the 3-month period after the introduction of the triage and signposting service, 2202 users obtained STI testing from Camberwell Sexual Health Service, 1266 users were signposted from this clinic after triage to the Web-based service, and 5362 used the Web-based service spontaneously. In addition, 175 people were triaged from another local sexual health service (Burrell St Clinic), who were excluded from subsequent analyses.

The introduction of the Web-based service and the triage and signposting service resulted in three different groups of service users (Table 2). For age, gender, and ethnicity, the clinic and the spontaneous online group showed differences in composition. The triage and signpost group was intermediate between the clinic and Web-based groups for age and ethnicity. For example, young people aged 16 to 19 years formed 8.89% (201/2261) of the clinic group, 4.55% (194/4262) of the spontaneous online group, and 6.2% (55/890) of the triage and signpost group.

The spontaneous Web-based group are most likely to be female: women formed 59.27% (1340/2261) of the clinic group, 64.43% (2746/4262) of the spontaneous Web-based group, and 46.7% (416/890) of triage and signpost group. Men who have sex with men formed a similar proportion of men using the clinic (30.4%, 251/825) and spontaneous Web-based (33.3%, 505/1516) but smaller proportion of triage and signposted (17.7%, 84/474), potentially reflecting higher probability of symptomatic infection or reported high risk behavior in the clinic population.

Both spontaneous Web-based service users and those registering at the clinic were encouraged to use clinic services if they had symptoms, and this is reflected in our data with 92.59% (3946/4262) of spontaneous Web-based users and 90.1% (802/890) of users who were triaged and signposted online classified as asymptomatic compared with 69.39% (1569/2261) of clinic users. 

**Table 1:** Changing pattern of activity at Camberwell Sexual Health Service (quarter 2 [Q2] and quarter 3 [Q3] 2016) and Web-based testing.

Description	Camberwell		SH:24 Web-based service	
	Q2, N or n (%)	Q3, N or n (%)	Q2, N	Q3, N
Total records (1 per person per day)	7662	6245	4439	7717
Included records (age≥16 years, <100 years, and no prisoners)	7593	6188	4439	7716
Total visits with any code (all users)	6946	5362	4439	7716
Total visits (Lambeth and Southwark residents)	5443	4172	4436	7073
Total STI^a^ tests (without complex intervention)	920 (16.90)	511 (12.25)	4436^b^	7073^b^
Total complex visits (with and without STI test)	3764 (69.15)	3123 (74.86)	0	0
Subtotal complex visits with STI test	2236	1691	0	0
Subtotal complex visit, no STI test	1528	1432	0	0
Other services used	759 (13.94)	538 (12.95)	0	0

^a^STI: sexually transmitted infection.

^b^Number of test kits sent out.

The test return rates within 6 weeks of test were higher for the spontaneous (70.51%, 3871/5632) than the “triage and signposting” groups (66.98%, 848/1266), which was significant (*P*=.01). A supplementary analysis comparing the demography of individuals who did and did not complete the testing process is shown in Multimedia Appendix 1. Women were less likely to complete tests, but otherwise, there were no significant differences between completers and noncompleters based on age, ethnicity, or sexual orientation, although the sample size is relatively small.

There were differences in positivity for any infection between the population that was seen in the clinic (7.70%, 174/2261), the *triage and signpost* users (6.4%, 57/890), and the *spontaneous* online users (4.58%, 195/4262). Patients diagnosed through Web-based testing were directed to clinics for management and partner notification in the same way as asymptomatic patients tested in the clinic. Any patients with HIV reactive tests are contacted to arrange confirmatory testing in the clinic.

**Table 2:** Characteristics of service users testing for sexually transmitted infections (STIs) via different pathways, July to September (quarter 3) 2016. A simple STI test includes chlamydia, gonorrhea, HIV, and syphilis.

Lambeth and Southwark residents (n=11,070)			Camberwell Sexual Health Clinic (n=4172)	SH:24 Web-based service (n=6898)^a^	
				Spontaneous online (n=5632)	Triage and signpost (n=1266)
Total visits, n			4172	5632	1266
Total tests ordered, n			2202	5632	1266
**Return rate, n**			N/A^b^		
	≤2 weeks, n (%)			3186 (56.57)	775 (61.22)
	>2 to ≤6 weeks, n (%)			785 (13.94)	73 (5.77)
	>6 weeks, n (%)			293 (5.20)	43 (3.40)
	Not returned, n (%)			1368 (24.29)	375 (29.62)
Total tests completed, n			2202	4262	890
Total tests completed plus STI diagnoses^c^, n			2261	—	—
**Age group, years, n (%)**					
	16-19		201 (8.89)	194 (4.55)	55 (6.2)
	20-24		472 (20.88)	1282 (30.08)	205 (23.0)
	25-29		508 (22.47)	1605 (37.66)	262 (29.4)
	30-34		365 (16.14)	650 (15.25)	146 (16.4)
	35+		715 (31.62)	531 (12.46)	222 (24.9)
**Gender, n (%)**					
	Female		1340 (59.27)	2746 (64.43)	416 (46.7)
	**Male**		921 (40.73)	1516 (35.57)	474 (53.3)
		Men who have sex with men	251 (27.3)	505 (33.31)	94 (19.8)
**Ethnicity, n (%)**					
	White		825 (36.49)	2850 (66.87)	461 (51.8)
	Mixed		178 (7.87)	353 (8.28)	93 (10.4)
	Asian		59 (2.61)	107 (2.51)	21 (2.4)
	Black or black British		939 (41.53)	768 (18.02)	270 (30.3)
	Other		208 (9.20)	105 (2.46)	32 (3.6)
	Missing or prefer not to say		52 (2.30)	79 (1.85)	13 (1.5)
**Symptoms, n (%)**					
	Asymptomatic		1569 (69.39)	3946 (92.59)	802 (90.1)
	Symptomatic		692 (30.61)	316 (7.41)	88 (9.9)
**Infection**					
	Positivity, any infection^d^, n (%)		174 (7.70)	195 (4.58)	57 (6.4)
	Chlamydia diagnoses, n		122	166	48
	Gonorrhea diagnoses, n		49	23	4
	Syphilis diagnoses, n		8	7	6
	HIV diagnoses, n		2	4	2

^a^175 referrals from Burrell St excluded.

^b^N/A: not applicable.

^c^with no test during visit.

^d^Separate STI diagnoses do not add up to the total as some individuals were diagnosed with multiple infections. Positivity is only indicated for any infection, not shown for each separate infection as the number of tests completed was different for each infection.

## Discussion

### Principal Findings

The key findings from this evaluation are that availability of Web-based testing increased the total volume of STI testing and increased the proportion of clinic visits which utilized a *complex* service requiring face-to-face clinical evaluation. Once established as an effective method of testing, the availability of Web-based STI testing resulted in a change in clinic policy to actively signpost clinic attendees to use the Web-based service, reflecting high trust in the Web-based service, but also high demands on the clinic services. The majority (70.30%, 890/1266) of clinic users who were signposted to the Web-based service successfully completed their test. Almost a third (29.62%, 375/1266) of clinic attendees did not successfully complete an STI test.

This evaluation shows that users can effectively be transitioned from Web-based to face-to-face services and that this introduces a new population to Web-based service use and changes the focus of clinic-based activity.

Changing patterns of disease and rising user expectations are increasing demands on health services [7,16]. Supported self-management is one element of the response and can be provided through Web-based health services. Web-based self-management services work best when integrated with and supported by face-to-face care [17]. This creates interfaces between Web-based and terrestrial services that sustain effective functioning of these emerging hybrid systems.

### Strengths and Limitations

Effective interaction with health services requires information and skills. This is acknowledged within sexual health services, with support for new service users such a young people to build capabilities for service access [18]. Self-management requires additional skills, and health services can support their development [19]. The triage and signposting intervention facilitated interaction with the Web-based service using strategies consistent with the literature on behavior change—making the transition easy, attractive, socially acceptable, and timely [20]. Tablets in the clinic and the test pack available immediately made the transition easy. Web-based services were attractive in that they avoided long waiting times in the clinic service. They were socially acceptable—with clinical staff promoting use and timely because they were offered at a time when users had identified a need for testing by visiting the clinic. By building capacity to self-manage STI testing, the service promotes a partnership approach to sexual health care delivered through the combined efforts of service providers and users [21,22]. However, nearly a third of those who were signposted to the Web-based service did not complete the testing process, and further service optimization is required to support shared decisions about clinic or Web-based service use that combine user preference, clinical guidance, evidence of efficient health service utilization, and that draw on a variety of user experiences. This work is in progress within this service using an agile approach with successive cycles of build-test-learn to optimize service design.

Across the whole sexual health economy, Web-based services increased total testing activity, suggesting a previously unmet need. During the study time frame, the testing volume in other service providers within the Southwark and Lambeth boroughs remained stable. Across England, there was a small increase in testing year on year (785,34—October 2014 to March 2015, increasing by 4% to 815,393—October 2015 to March 2016), which is in contrast to a 18.47% increase observed in Lambeth and Southwark. Before the introduction of triage and signposting, early adopters of Web-based STI testing were more likely to be women, aged 20 to 35 years, and of white ethnicity [23]. The triage and signposting intervention expanded the Web-based testing population compared with the group accessing via Web-based testing spontaneously. The residual population of clinic users were then more likely to have complex needs. Over a quarter of users who were signposted to the website (ordering a test with support in the clinic) did not complete the self-sampling and return a sample for testing. Women were less likely to complete the test in this group than men (Multimedia Appendix 1). A similar proportion of individuals using the Web-based service spontaneously also did not complete their tests. Noncompletion of tests for users in the triage and signposting group suggests possibility of missed diagnoses; however, it is not possible to ascertain whether these individuals were tested in other settings, for example, general practitioner or remained untested.

Triage and signposting changed patterns of unmet need in the sexual health economy studied. The continued increase in testing volume did not saturate during the study period, suggesting a continued unmet need in this population. The users who were previously turned away were advised to try another clinic or try the same clinic at a different time. The triage and signposting system offers these users a new option.

The intervention responds to two explicit objectives for the NHS: (1) to increase efficient use of resources and (2) to deliver user-centered care [7]. The evaluation suggests that it is partially successful on both counts. By focusing face-to-face clinical resource on complex need and shifting simple activity to supported self-management, it improves the efficiency of the sexual health economy. By building the capacity to use Web-based services, it offers an additional choice for STI testing that is potentially more convenient and more accessible [9,24].

Finally, this evaluation underlines the importance of research on the interfaces between Web-based and face-to-face services within the context of a whole sexual health economy. It suggests that users may be willing and able to move between Web-based services and clinic-based services with further research needed on how and why users transition between service modalities.

### Implications

This paper reports on an innovative service evaluated in a timely way to inform service development. The use of routinely collected data collected offers consistent information on all attendances at all services in the area.

This paper evaluates a clinic-led service improvement in a complex and changing environment rather than a planned research intervention. Some important questions such as the subsequent STI testing behavior of those who did or did not transition to Web-based services could not be answered with routinely collected data. We were unable to compare rates of treatment and partner notification with the routine data; however, positive patients were managed in the same way following diagnosis according to relevant clinical guidelines. Some assumptions such as the use of microscopy as a proxy for symptoms among clinic users are not substantiated. Additionally, some variables were self-reported in clinic data and could be missing where online users were required to select gender and sexual orientation to determine which test kits to send out.

Further research is required to investigate the long-term changes in service use behaviors, for example, the choice of service for subsequent STI testing, the reasons that those signposted online do not make the transition, improved strategies for triage and signposting so that those signposted are more likely to shift online, and the cost effectiveness of the shift in activity for sexual health economies.

## Appendix

Multimedia Appendix 1 Further data on coding, additional analysis, and definition of positivity.

## Abbreviations

GUM: genitourinary medicine
NHS: National Health Service
STI: sexually transmitted infection
Q1: quarter 1
Q2: quarter 2
Q3: quarter 3
