# Supplementary material for: Web-Based Activity Within a Sexual Health Economy: Observational Study
Source: J Med Internet Res. 2018 Mar 7;20(3):e74. doi: 10.2196/jmir.8101 (PMC5863011; doi:10.2196/jmir.8101)
Supplement: Multimedia Appendix 1 [file jmir_v20i3e74_app1.pdf]

Multimedia Appendix 1

Table S1: GUMCAD codes available from https://www.gov.uk/government/uploads/system/uploads/attachment_data/file/420625/GUMCADv2_SHHAPT_Code_Look-up.pdf

Public Health England

SHHAPT Code Look-Up

Sexual Health & HIV Activity Property Type Codes - Summary of Definitions

DIAGNOSIS, CONDITION OR DISEASE		SHHAPT
**Balanitis / vaginitis / vaginosis** (other causes)		C6C
**BV* & anaerobic balanitis**	*Bacterial vaginosis	C6B
**Candidosis**		C7
**Cervical cytology**	Minor abnormality	P4A
	Major abnormality	P4B
**Chancroid**		C1
**Chlamydia**		C4
**Donovanosis**		C3
**Gonorrhoea**		B
**Hepatitis**	A - acute infection	C15
	B - 1st diagnosis	C13
	C - 1st diagnosis	C14
**Herpes** (genital)	1st episode	C10A
	Recurrent episode	C10B
**HIV**	Known positive	H
	New diagnosis	H1
	New diagnosis – acute infection	H1A
	New diagnosis – AIDS defined	H1B
	HIV related care	H2
**LGV**	Lymphogranuloma venereum	C2
**Molluscum contagiosum**		C12
**Mycoplasma genitalium**		C16
**NSGI**	Non specific genital infection	C4N
**Ophthalmia neonatorum**		C5B

DIAGNOSIS, CONDITION OR DISEASE		SHHAPT
**Other conditions requiring treatment**		D2B
**Pediculosis pubis**		C9
**PID & epididymitis**		C5A
**Pregnant**	1st trimester (1-12 weeks)	PR1
	2nd trimester (13-28 weeks)	PR2
	3rd trimester (29 weeks - full term)	PR3
**Scabies**		C8
**Sexual assault**	Acute - medical exam within 7 days	40
	Non-acute - medical exam more than 7 days	41
**Shigella**	Flexneri	SG1
	Sonnei	SG2
	Other / unspecified	SG3
**Syphilis**	Primary	A1
	Secondary	A2
	Early latent	A3
	Cardiovascular	A4
	Neurosyphilis	A5
	Other late and latent	A6
	Congenital	A7A
**Trichomoniasis**		C6A
**UTI**	Urinary tract infection	D2A
**Warts** (genital)	1st episode	C11A
	Recurrent episode	C11D

SERVICES PROVIDED		SHHAPT
**Cervical cytology done**		P4
**Contraception**		P3
**Hepatitis B immune**		P2I
**Hepatitis B vaccination**	1st dose	P2A
	2nd dose	P2B
	3rd dose	P2C
	4th dose	P2D
	Booster	P2E
**HPV vaccination**	1st dose	W1
	2nd dose	W2
	3rd dose	W3
**No service and/or no treatment required**		D3
**Partner notification**	Initiated*	PN
	Chlamydia contact	PNC
	Gonorrhoea contact	PNG
	HIV contact	PNH
	NSGI contact	PNN
	PID / epididymitis contact	PNP
	Syphilis contact	PNS
	Trichomoniasis contact	PNT

SERVICES PROVIDED			SHHAPT
**Patient type**	Prisoner		Z
	Sex worker		SW
	Sexual & reproductive health		SRH
**PEPSE**	Post exposure prophylaxis sexual exposure		PEPS
**Referral type**	from NCSP		REF1
	to GUM (Level 3)*		REF2
	from home testing / sampling		REF3
**Testing -**	**HIV**	HIV antibody test	P1A
		HIV test offered & refused	P1B
		HIV test not appropriate	P1C
	**STIs**	Chlamydia only	T1
		Chlamydia & gonorrhoea	T2
		Chlamydia, gonorrhoea & syphilis	T3
		Chlamydia, gonorrhoea, syphilis & HIV	T4
		HSV (herpes simples virus)	T5
		Hepatitis A / B / C	T6
		Syphilis & HIV	T7
		Self sampling (no HCW consultation)	T8
		STI tests not required	T9
		Rapid testing (same day results)	T10
		Microscopy	TS
		3 site testing	TT

SUFFIX NAME	VALID SHHAPT CODE	SUFFIX
**Diagnosed previously elsewhere**	A1, A2, A3, A4, A5, A6 A7A, B, C4, C6A, H1, H1A, H1B	X
**Medication given***	B, C4, C10A, C10B, C11A, C11D	M

SUFFIX NAME	VALID SHHAPT CODE	SUFFIX
**Pharyngeal infection**	B, C2, C4	O
**Quadrivalent HPV vaccine**	W1, W2, W3	Q
**Rectal infection**	B, C2, C4, C4N	R

*For use in Level 2 (non-GUM) services.

© Crown copyright 2015

Table S2: Use of GUMCADv2 codes to categorise sexual health attendances

	**GUMCAD codes**			
Label	SHHAPT	SHRAD	Contraception	Description
Simple service use e.g. STI testing / contraception / advice				
**STI test (simple)**	T1, T2, T3, T4, TT, T8, T7, P1A			Chlamydia, gonorrhoea, syphilis, HIV, Syphilis, self-sampling
**Other (simple)**	P3, A1, A2, A3, A5, A6, B (O,R), C4 (O,R), H, H1, H1B, H1A, D3, PN, PNC, PNG, PNS, P1B, P1C, P3, SW,SRH, T9	1	5, 6, 7, 8, 12	Simple contraception<br>STI diagnosis: (Chlamydia, gonorrhoea, syphilis, HIV)<br>Advice / health promotion / partner notification
Complex visit (requiring face-face clinic visit or physical exam/intervention)				
**Complex visit**	T5, T6, T10, TS, C6C, C6B, C7, C1, C3, C15, C13, C14, C10A(M), C10B(M), C2, C12, C16, C4N(R), C5B, D2B, C9, C5A, C8, SG1, SG2, SG3, A7A, C6A, D2A, C11A(M), C11D(M), C2R, C4N(R), C6A, P4A, P4B, P2A, P2B, P2C, P2D, P2E, W1(Q), W2(Q), W3(Q), PEPS, H2, P4, (P2I), PR1, PR2, PR3, 40, 41, PNN, PNT, REF1, REF2, REF3	34,19, 20, 21, 22, 23, 35, 36, 29L, 29C, 11, 14, 15, 16,17, 18, 24, 25,26, 37, 38, 39, (29G, 29D), 4, 5, 6, 7, 8, 9, 10, 2, 3, 27, 28, 29, 12, 13, 30, 31, 32, 33,	1, 2, 3, 4, 9, 10, 11, 13	Other STI test: HSV, Hepatitis (A,B or C), Microscopy, symptoms/ Diagnosis/ conditions other than above, Other contraception e.g intrauterine device insertion or removal, Other interventions /vaccination /surgical, Abortion & related, Pregnancy & related, Other services e.g. Sexual assault, referral,to pychosexual counselling

Equation E1: Positivity definition

STI diagnosis was extracted based on the GUMCAD codes. Positivity is calculated as follows

Positivity = Number positive/(Number positive + Number negative)

For SH24 data each line includes the STI test and the result of that test, so positivity calculation is straightforward. In clinic settings positive results and treatment codes are assigned to the original test record, however diagnosis codes may also appear without associated test records (e.g. if an individual has been diagnosed elsewhere). This could include individuals who had been diagnosed via the online service and referred to clinic for treatment. We therefore calculate clinic positivity as number of positive diagnoses/total test records.

Supplementary analysis

Supplementary analysis of those who did and did not complete online testing following triage and signposting in Q3 2016.

Complete information on age, gender, sexual orientation and ethnicity were available for 1240/1266 individuals who were directed to online testing via triage and signposting. We used logistic regression to identify any differences is completion rate according to these factors.

Table S3 Supplementary comparison of individuals who received triage and signposting to use online testing who did and and did not complete the testing process

	Completed test (n=1,240)	Adjusted Odds Ratio (AOR)	p value	95% Conf. Interval	
Gender	Male	REF			
	Female	0.66	.002	0.51	0.86
Ethnic group	White	REF			
	Mixed	1.02	0.93	0.67	1.55
	Asian	1.61	0.35	0.60	4.36
	Black or black British	0.97	0.83	0.73	1.29
	Other	0.83	0.55	0.45	1.53
Sexual orientation	Heterosexual	REF			
	Homosexual	0.71	0.14	0.46	1.12
	Bisexual	1.50	0.18	0.83	2.71
	age	1.00	0.87	0.99	1.02
	Constant	2.94	0	1.82	4.76

Women appeared less likely to complete the testing process than men (AOR 0.66, CI 0.51 to 0.86), but there was no statistically significant effect of age, ethnic group or sexual orientation.
